# Enigmatic H_2_- and CH_4_-rich hydrothermal plumes at the ultramafic-hosted Lucky B site, 81°N on Lena Trough, Arctic Ocean

**DOI:** 10.1038/s41598-025-19746-5

**Published:** 2025-10-14

**Authors:** Elmar Albers, Felix Genske, Jeffrey S. Seewald, Maren Walter, Jonathan Mette, Gunter Wegener, Massimiliano Molari, Christopher Klaembt, Luigi Gallucci, Tea Isler, Lilian Böhringer, Jessica N. Fitzsimmons, Shelby Gunnells, Vera Schlindwein, Christopher R. German

**Affiliations:** 1 Department of Geology & Geophysics, Woods Hole Oceanographic Institution, Woods Hole, USA; 2 Institute for Mineralogy, University of Münster, Münster, Germany; 3 Department of Marine Chemistry & Geochemistry, Woods Hole Oceanographic Institution, Woods Hole, USA; 4 Institute of Environmental Physics, University of Bremen, Bremen, Germany; 5 MARUM – Center for Marine Environmental Sciences, University of Bremen, Bremen, Germany; 6 Max Planck Institute for Marine Microbiology, Bremen, Germany; 7 Faculty of Geosciences, University of Bremen, Bremen, Germany; 8 Section of Geophysics, Alfred Wegener Institute Helmholtz Centre for Polar and Marine Research, Bremerhaven, Germany; 9 Deep-Sea Ecology and Technology, Alfred Wegener Institute Helmholtz Centre for Polar and Marine Research, Bremerhaven, Germany; 10 Department of Oceanography, Texas A&M University, College Station, USA; 11 Present Address: Section of Geophysics, Alfred Wegener Institute Helmholtz Centre for Polar and Marine Research, Bremerhaven, Germany; 12 Present Address: IMC International Marine Centre, Oristano, Italy

**Keywords:** Marine chemistry, Element cycles, Microbial communities

## Abstract

Tectonic uplift of mantle rocks along slow- and ultraslow-spreading mid-ocean ridges facilitates diverse styles of hydrothermal circulation. Here, we report on Lucky B, an ultramafic-hosted hydrothermal field on the ultraslow-spreading Lena Trough at 81°N in the ice-covered Arctic Ocean. At the seafloor we observed diffuse, metal-poor fluid discharge with abundant vent fauna alongside sites of massive sulfide deposits and hydrothermal chimneys, extending laterally over at least 1.9 km. The overlying water column exhibited two geochemically distinct plumes, the stronger of which showed strong redox and particle anomalies. We hence identify Lucky B as ‘black smoker’-type system featuring distinct styles of venting from several major fluid sources. The strongest plume also contained high concentrations of dissolved hydrogen (H_2_) and methane (CH_4_), distinguishing Lucky B from other ultramafic-hosted systems that primarily emit serpentinization-derived H_2_. Low H_2_/CH_4_ ratios and high CH_4_ relative to dissolved Mn suggest an involvement of sediment in the subseafloor fluid–rock reactions. Our analysis of the plume microbiology revealed abundant chemoautotrophs that use primarily hydrothermal H_2_ and sulfide as energy sources. Collectively, these findings reveal multifaceted hydrothermal venting at Lucky B, driven by geological and biogeochemical processes in the subseafloor and extending into the Arctic Ocean water column.

**Supplementary Information:**

The online version contains supplementary material available at 10.1038/s41598-025-19746-5.

## Introduction

Where uplifted towards the seafloor, ultramafic rock from the Earth’s mantle reacts with seawater during hydrothermal circulation. This ‘serpentinization’ process produces molecular H_2_ which, upon release into the overlying ocean, establishes redox disequilibria. Ultramafic-influenced submarine hydrothermal systems are of particular interest because there, high levels of serpentinization-derived H_2_ can enable the abiotic synthesis of key organic species in addition to elevated concentrations of dissolved CH_4_^1–3^. The same H_2_-rich hydrothermal systems can also provide particularly high levels of metabolic energy to sustain chemosynthetic ecosystems^4,5^.

The uplift of ultramafic rock to shallow crustal levels—or to the seafloor itself—is widespread at ultraslow-spreading mid-ocean ridges, which constitute some 25% of the global ridge length^6^. These ridges are marked by extensional faulting with discrete areas of either elevated or sparse magmatism^7^, conditions that promote deep seawater penetration and circulation within the lithosphere^8,9^. Accordingly, hydrothermal plume signals at ultraslow-spreading ridges are up to three times more frequent than predicted by models based solely on axial magmatic heat flux^10,11^. This discrepancy from faster-spreading ridge models^12^ likely reflects the broader range of geologic settings that support more diverse venting styles along ultraslow-spreading ridges^13–17^.

Along the ultraslow-spreading Gakkel Ridge in the high Arctic, at least nine distinct hydrothermal plume sources have been discovered, establishing proof that this ridge hosts abundant hydrothermal activity^18,19^. Gakkel Ridge exploration is, however, particularly difficult due to its remoteness and perennial sea ice cover, so that only two sites, ‘Aurora’ and ‘Polaris’, have been tracked to their seafloor sources to date. Both are characterized by fluids with elevated H_2_ and CH_4_ concentrations, typically linked to serpentinization of ultramafic rock^16,17,20–22^. Yet, only *mafic* rocks have been observed at the seafloor at both sites^16,17^. The ultramafic lithologies required to produce the chemical signatures detected must therefore lie in the subseafloor along the flow paths of the hydrothermal convection cells and, hence, beneath relatively thin veneers of basalt relative to the typical ~ 6 km thick crust found at faster-spreading ridges. Variably thick volcanic crust has been recognized as a key feature of ultraslow-spreading ridges^8,9^ and on Gakkel Ridge, seismic experiments indicated that thicknesses reach only ~ 3–4 km in areas distant from major volcanic centers^23,24^.

**Fig. 1:**
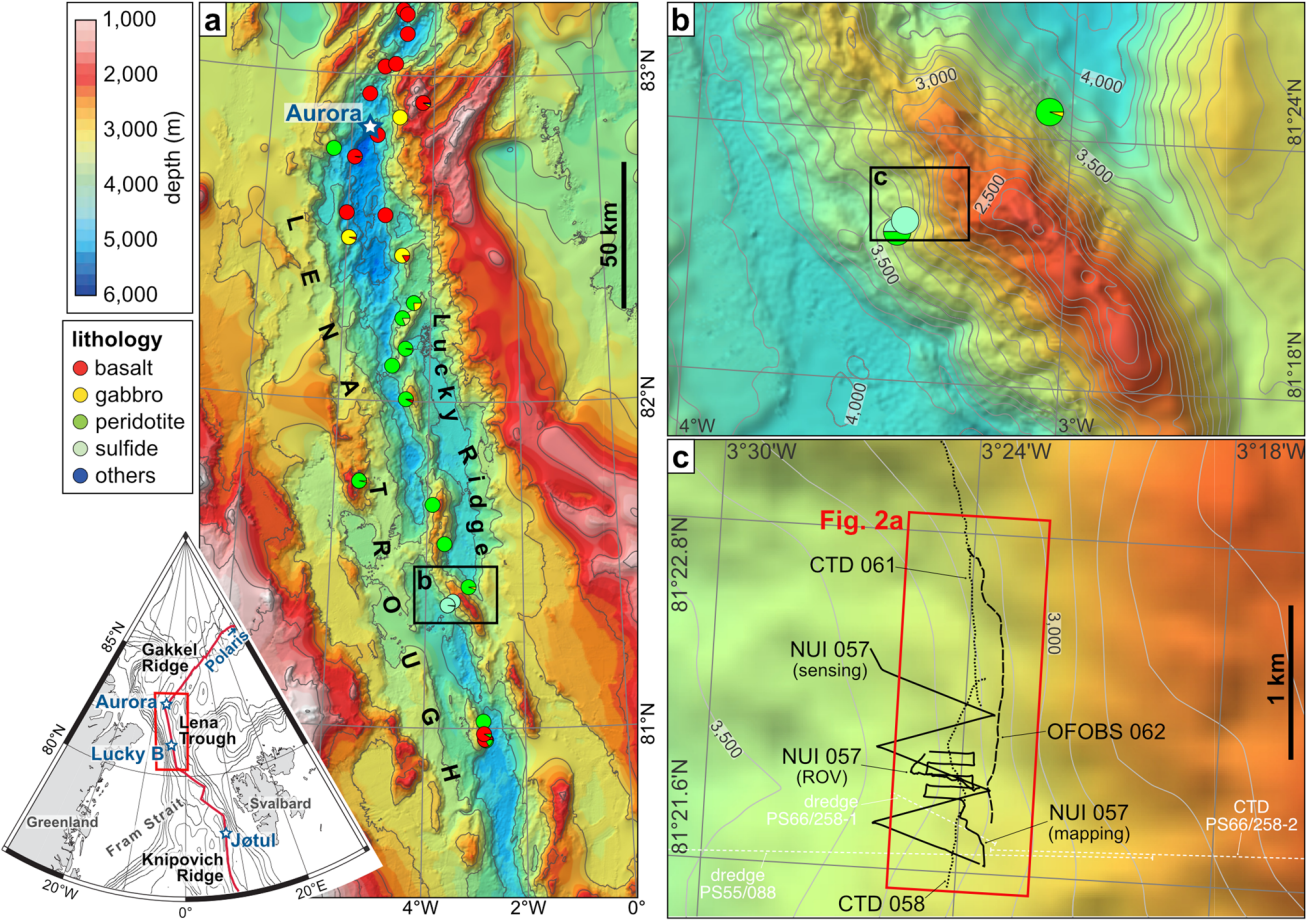
Bathymetric maps of the study area in the Lena Trough. **(a)** Overview maps. Inset shows the greater Fram Strait area with locations of active seafloor spreading centers. The nearest-known hydrothermal systems to Lucky B, Aurora and Jøtul, are also indicated; Polaris, the only other known system in the high Arctic, is located further to the northeast on Gakkel Ridge, at 86°58ʹN, 55°45ʹE. The red rectangle marks the position of the larger map of the Lena Trough, revealing the extent of the ‘Lucky Ridge’ in Lena Trough’s central rift axis. Pie charts represent rock lithologies derived primarily from dredge statistics from R/V Polarstern expeditions PS55 and PS66^29,30^. Maps made with QGIS Geographic Information System version 3.44.0 (https://qgis.org), using the 200 m resolution IBCAO v5.0 bathymetry^92^ and a polar stereographic projection. **(b)** Detailed map of the section of Lucky Ridge studied, as marked by the black rectangle in (a). The Lucky B hydrothermal field is located on the western slope of the Lucky Ridge. **(c)** Close-up of the rectangle in (b) showing the tracks of our CTD, OFOBS, and NUI deployments at Lucky B. A synthesis of the hydrothermal observations from these deployments is shown in Fig. 2. Dredge and CTD tracks from expeditions PS55 (PS55/088) and PS66 (PS66/258–1 and PS66/258–2) that led to the initial discovery of the Lucky B site are also indicated. Bathymetry in (b) and (c) were acquired during expedition PS137^33^ using the ship’s multibeam system.

Even less is known about hydrothermal activity in Gakkel’s southward continuation, the Lena Trough, which laterally offsets the Greenland shelf from the Yermak Plateau (Fig. 1). Lena Trough is characterized by oblique spreading with northwest–southeast-oriented extension at ~ 13 mm/yr^25,26^. Seafloor magnetic data, seismicity, and rock sampling suggest it undergoes primarily tectonic extension with sparse magmatism^26,27^. This is supported by a series of steeply dipping *en echelon* peridotite blocks forming the so-named ‘Lucky Ridge’^26^ (Fig. 1a). In 1999, evidence for geologically *recent* hydrothermal activity was established towards the base of Lucky Ridge’s western slope, at ~ 3,300 m depth at ~ 81°22ʹN, in the form of massive sulfide deposits recovered, serendipitously, during petrologic dredging^28,29^ (Fig. 1c). These findings revealed the ‘Lucky B’ hydrothermal field^28^. A return to the location in 2004 yielded additional massive sulfide crusts and chimney materials, and a single water column profile, acquired using a conductivity–temperature–depth (CTD) unit, detected temperature anomalies at ~ 3,250 m water depth, confirming *ongoing* venting at Lucky B^30^. Lucky B is the first site found along the Arctic ridge system where ultramafic rock outcrops at the seafloor, and one of less than ten such sites known globally^31,32^.

Here, we present results from R/V Polarstern cruise PS137^33^ during which we investigated the hydrothermal plume system overlying the Lucky B site and obtained new observations at the seafloor. Our data reveal ongoing ultramafic-influenced venting that discharges H_2_- and CH_4_-enriched fluids into the Arctic Ocean, expanding our understanding of the diverse hydrothermal activity along Earth’s slowest-spreading ridge system in the high Arctic.

## Results and discussion

### Distinct types of hydrothermal plumes at Lucky B

**Fig. 2:**
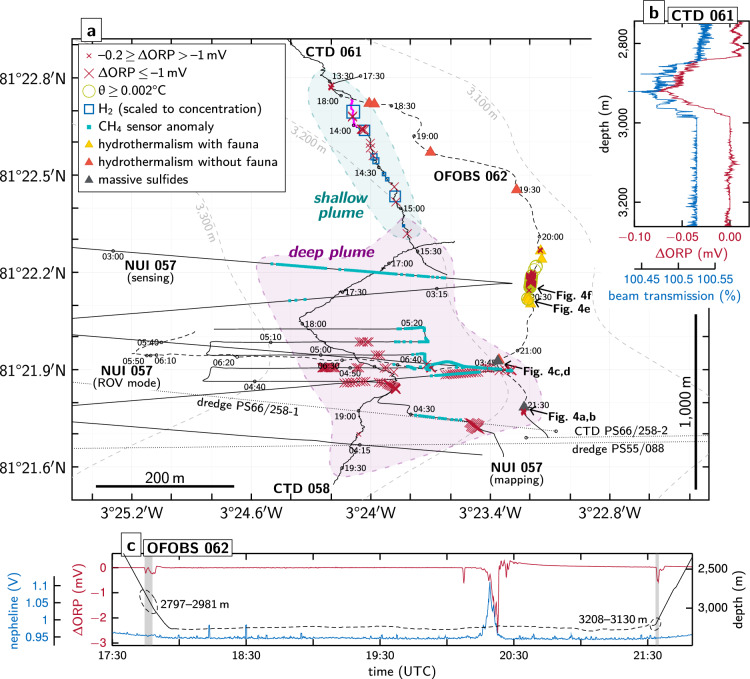
Synthesis figure summarizing water column and seafloor imaging results. (a) Deepest hydrothermal signals were recorded just above the seafloor, where NUI operated in ROV mode detected anomalies in ORP and CH_4_ near 81°21.9ʹN (Supplementary Fig. S1) and the OFOBS system revealed visual and sidescan sonar evidence of hydrothermally active seafloor, together with co-registered ORP anomalies, between 3,100–3,200 m depth (Figs. 2c and 4, Supplementary Figs. S4 and S5). In the overlying water column, NUI’s mapping and sensing surveys and our CTD casts intercepted two distinct non-buoyant plumes: a deep plume at > 3,000 m depth with weak ΔORP and a shallow plume at ~ 2,800–3,000 m with strong ΔORP and elevated optical backscatter values as well as high H_2_ and CH_4_ concentrations (Figs. 2b, 3 and 5, Supplementary Figs. S2 and S3). Hydrothermal signals from these CTD and NUI operations extended farther south as compared to our seafloor imaging surveys, where they converged with the locations at which massive sulfide material and water column hydrothermal anomalies had previously been observed (dotted lines representing track lines from prior expeditions^29,30^), highlighting both the wide lateral extent and the decade-long activity of the Lucky B hydrothermal field. Shown tracks are cropped to ≥ 2,000 m depth. Dashed track lines indicate positions of NUI and OFOBS directly above the seafloor. Horizontal scale bar represents 200 m at 81°22.2ʹN; note that the north–south distance between 81°21.6ʹN and 81°22.8ʹN is ~ 2,200 m. **(b)** Depth profile from a single upcast of CTD 061 (at ~ 13:51–13:56 UTC near 81°22.7ʹN, indicated in (a) as a thicker pink line), displaying coinciding negative ORP (30 s averages) and beam transmission anomalies diagnostic of a fresh, particle-laden plume. **(c)** Time-series MAPR record from station OFOBS 062 showing pronounced ΔORP (30 s averages) and positive temperature anomalies at ~ 20:20 UTC, coincident with images of abundant, highly localized fauna (Fig. 4f, e). Gray bars indicate OFOBS intersections with the shallow and deep plumes at the start and end of the deployment, respectively. Dashed line marks OFOBS towed few meter above the seafloor.

We conducted a series of water column surveys and seafloor observations at the Lucky B site, which provided multiple independent lines of evidence for ongoing hydrothermal activity. These results are summarized in Fig. 2; details on the operational strategy are included in the Supplementary Information.

**Fig. 3:**
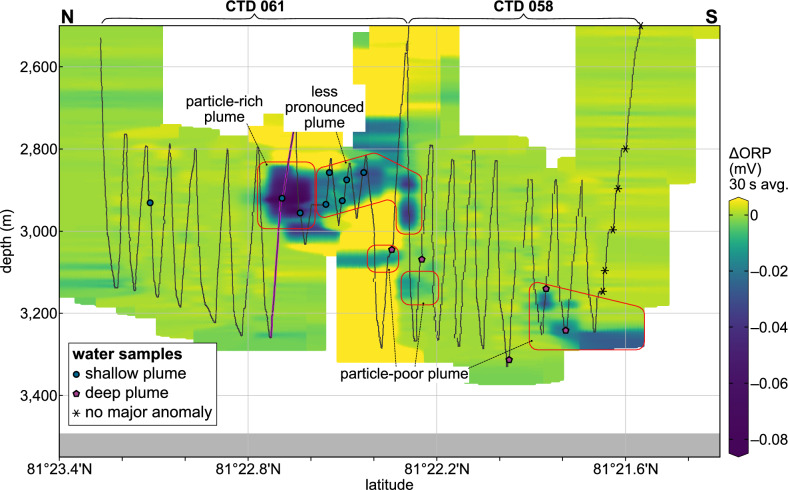
Water-column distribution of the Lucky B plumes. Shown are ΔORP signals (30 s averages) recorded with the CTD unit, interpolated and projected on a north–south profile across the study area. Anomalies indicative of particle-rich venting were detected in the shallow plume at ~ 2,800–3,000 m water depth and a deep particle-poor plume was recorded at >3,000 m depth, with strongest signals towards the profile’s southern end; see text for discussion. We also intersected these shallow and deep plumes when lowering the OFOBS device to the seafloor and heaving it back up, respectively (cf. Fig. 2c). Also shown are positions of water samples taken with the CTD unit. Black dotted lines mark projected depth of CTD; pink thicker line at ~ 81°22.7ʹN indicates vertical profile presented in Fig. 2b. Section created using Ocean Data View^93^.

Guided by hydrothermal signals from the single CTD cast in 2004^30^, we identified two distinct non-buoyant plumes in the water column above the western slope of Lucky Ridge: (i) a shallow plume between ~ 2,800–3,000 m depth and (ii) a deeper plume at ~ 3,000–3,200 m (Figs. 2a and 3). The deeper, more southerly plume extended between ~ 81°21.7ʹN to 81°22.3ʹN and was characterized by anomalies in oxidation–reduction potential (ORP) and CH_4_, with minimal turbidity (Figs. 2a and 3, Supplementary Figs. S1 and S2), as recorded by the hybrid remotely operated vehicle (ROV) NUI and during CTD cast 058. NUI encountered the strongest signals in the eastern part of its survey area, near the ~ 3,250 ± 50 m depth contour, with 30 s-averaged ΔORP down to –0.043 mV (Fig. 2a, Supplementary Fig. S1). During the subsequent CTD 058, the most pronounced ΔORP values occurred at and below ~ 3,150 m, reaching –0.048 mV (Figs. 2c and 3, Supplementary Fig. S2). These anomalies were observed as the CTD passed across the dredge tracks from 1999 and 2004 and through the same 3,200–3,300 m depth interval where plume signatures were recorded in 2004^29,30^. No evidence of particle enrichment was detected in the deep plume.

The shallower plume was discovered further north, between ~ 81°22.3ʹN and 81°22.8ʹN (Fig. 2a). Pronounced redox anomalies, with 30 s-averaged ΔORP reaching –0.089 mV, and elevated optical backscatter were recorded during the CTD 061 survey and the initial downcast of CTD 058 (Figs. 2b, c and 3, Supplementary Fig. S3). From north to south, the shallower plume gradually shoaled, with anomalies persisting during the final upcast of CTD 061 at depths similar to those observed in CTD 058 (Fig. 3). South of ~ 81°22.6ʹN, a deeper set of anomalies appeared, deepening from ~ 3,000 m to ~ 3,100 m over the remainder of CTD 061 (Fig. 3). The strongest backscatter signals, however, were observed ~ 500 m farther south, near 81°22.4ʹN, where targeted water column sampling also revealed high concentrations of dissolved H_2_ and CH_4_ (see below).

The recurrence of a non-buoyant plume at the same depth and location as detected in 2004^30^ indicates sustained hydrothermal activity at Lucky B over at least two decades. Located atop a peridotite ridge, Lucky B’s geologic setting aligns with tectonically controlled systems such as TAG and Rainbow on the Mid-Atlantic Ridge, both of which have remained active over millennial timescales^34,35^. The distribution of the two plumes detected—adjacent to, but deeper than, the summit of the Lucky Ridge—implies they are influenced by topographic steering^36^.

The observed ORP anomalies and particle enrichment within the shallow plume are consistent with high-temperature ‘black smoker’-type venting. In contrast, in situ sensor data for the deeper plume revealed elevated CH_4_ and other chemically reduced species but without any accompanying particle enrichments of the kind typically associated with black smoker systems, suggesting venting temperatures too low to mobilize substantial amounts of dissolved metals, i.e., below ~ 300 °C^37^. These contrasting signatures indicate the presence of at least two distinct hydrothermal sources across the ~ 2 km extent of our surveys: a high-temperature vent site in the north and a particle-poor source farther south. Supporting this interpretation, the shallow and deep plumes lie on different isopycnal surfaces (Supplementary Fig. 2e), reflecting hydrothermal fluids with distinct physical properties^38^.

Further evidence for multiple fluid sources at Lucky B comes from subsequent discoveries aboard the Norwegian ship R/V Kronprins Haakon^39^. Arriving on station just before we completed our studies, and informed by our discoveries, the Norwegians conducted ROV dives that located a series of actively venting black smoker chimneys (https://gonortharctic.no/). However, those chimneys, named ‘Ultima Thule’, were located at a shallower seafloor depth than the hydrothermal plumes reported here and therefore cannot be the source of either the deep or the shallow plume described in this study. Instead, the Ultima Thule vents likely feed a separate, even shallower plume—supporting the presence of at least three distinct major hydrothermal sources at the Lucky B hydrothermal field.

### Widespread active and extinct seafloor hydrothermal activity

At the seafloor, we conducted a west–east geological transect deploying NUI in ROV mode (Fig. 2a), which revealed a predominantly sediment-covered terrain with occasional peridotite exposures. These outcrops showed localized discoloration and minor hydrothermal precipitates that became increasingly abundant upslope. No visible fluid discharge was observed. However, just after departing the seafloor east of 3°23.8ʹW at 3,190 m depth, NUI detected pronounced ΔORP values exceeding –2 mV, accompanied by elevated CH_4_ concentrations (Supplementary Fig. S1).

**Fig. 4:**
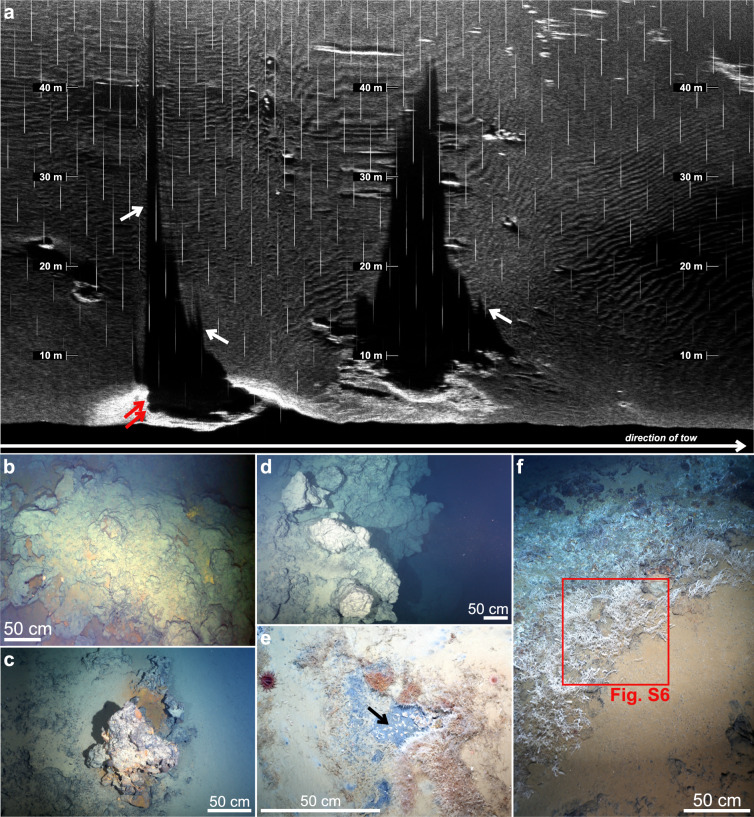
Evidence of hydrothermally active seafloor documented during the OFOBS survey. **(a)** Sidescan sonar image acquired along the OFOBS trackline. Circular features (red arrows) and elongated, spiky shadows (white arrows) suggests the presence of hydrothermal chimneys located just beyond the visual range of the OFOBS cameras. Also note the relatively smooth, sedimented seafloor. **(b)** Seafloor photograph taken at the site where the sidescan sonar depicted chimney structures, as shown in (a). **(c, d)** Traces of (focused) venting, including massive sulfide deposits exhibiting rust-orange weathering and remnants of extinct hydrothermal chimneys. **(e, f)** Dense hydrothermalism-associated fauna observed in a region exhibiting pronounced anomalies in ORP, optical backscatter, and temperature near the seafloor (cf. Fig. 2a, c). Panel (**e**) highlights amphipods (arrow), tubeworms, and red anemones within a pocket of presumed diffuse hydrothermal discharge. The white fibrous organisms in (**e**) and (**f**) are likely tubeworms covered in chemosynthetic bacteria, resembling siboglinid tube worms and microbial filaments found at the nearby Jøtul site^40^. A close-up is shown in Supplementary Fig. S6; note, however, that we did not collect physical specimens of the fauna to validate the seafloor images. Also note the moderately sedimented seafloor with localized exposures of bedrock, likely consisting of variably serpentinized ultramafic rocks. Refer to Fig. 2a for image locations.

We also carried out a north–south seafloor imaging survey using the OFOBS deep-tow system along the 3,150 ± 50 m depth contour, where the strongest plume signals had been identified (Fig. 2a). As OFOBS was lowered to the seafloor, in situ sensors mounted on its frame recorded ORP and optical backscatter anomalies that mirrored those first detected at nearly the same location and depth during CTD 061. Upon retrieval, the same sensors detected deeper plume signals consistent with those identified during CTD 058 (Fig. 2c, Supplementary Fig. S4). Seafloor imagery revealed a sedimented landscape punctuated by occasional peridotite outcrops, as well as multiple indicators of hydrothermal activity along the ~ 1.9 km track. These visual indicators frequently coincided with ORP and/or temperature anomalies recorded by the OFOBS sensor package (Fig. 2a, c), signaling that there was active seafloor fluid flow in close proximity to our ~ 3 × 5 m down-looking field of view. At ~ 3,200 m depth near 81°21.9ʹN, where NUI had recorded the strongest ORP and CH_4_ anomalies, the OFOBS sidescan sonar revealed a series of chimney-like structures at a lateral range of ~ 5–10 m to the east, just beyond the view of its down-looking cameras (Fig. 4a, Supplementary Fig. S5 and Table S1). Within the co-located seafloor images, we could clearly observe polymetallic massive sulfide mounds with rust-orange weathering (Fig. 4b). Later during the deployment, towards the area where sulfides had been dredged during earlier expeditions^29,30^, we observed further and extensive massive sulfide deposits including clearly recognizable chimney morphologies (Fig. 4c, d). Slightly upslope, more towards the 3,100 m contour line (at ~ 18:15–20:30 UTC; Fig. 2a), we documented multiple distinct patches of discolored orange sediment consistent with hydrothermal staining. Near 81°22.2ʹN, the deep-tow system traversed across an area of remarkably dense biomass, not imaged anywhere else. There, an unusually dense aggregation of benthic organisms was observed including invertebrates, amphipods, and elongated organisms reminiscent of siboglinid tubeworms (Fig. 4e, f, Supplementary Fig. S6). At the same time that these photographs were taken, OFOBS’ in situ sensors recorded the strongest signals indicative of hydrothermal flow from the entire study of the Lucky B area, including pronounced ΔORP peaks (exceeding –2 mV) and increases in bottom water temperature (θ > 0.004 °C; Fig. 2c, Supplementary Fig. S4). We did not, however, observe any traces of high-temperature venting or hydrothermal chimney structures in the close vicinity of this site. Instead, our observations could be explained by the presence of diffuse hydrothermal flow sustaining a thriving chemosynthetic ecosystem there.

In sum, our findings—paired with the recovery of massive sulfides from the area in 1999 and 2004^28,29^—indicate that hydrothermal activity extends over a considerable range along the western flank of Lucky Ridge. Seafloor images and anomalies detected by water column sensors indicate the presence of multiple, discrete, active and inactive sources of seafloor fluid discharge, encompassing a diverse array of venting styles. These styles include diffuse lower-temperature fluid flow together with sites of active and extinct high-temperature black smoker venting. A similarly broad range of vent types has recently been documented from the Jøtul hydrothermal field—the nearest known vent site to the south—which is located on the similarly oblique-spreading Knipovich Ridge (Fig. 1). At Jøtul, fault-controlled venting supports emissions that range from clear fluids at just 8 °C to black smoker vents as hot as 317 °C^40^. In contrast to Lucky B, however, Jøtul only produces a single hydrothermal plume, likely because nearby low-temperature fluid emissions are entrained and mixed into the buoyant plume from the high-temperature vent. The presence of two distinct plumes at Lucky B, by comparison, suggests two physically separate vent sources. The vent fauna observed at Lucky B (Fig. 4e, f) bear a close resemblance to the chemosynthetic communities at Jøtul and also at Loki’s Castle, a vent site at the Mohns–Knipovich junction, where siboglinid tubeworms and microbial mats have been identified^40,41^. Notably, no such tubeworms were observed at the closest-known vent site to the north, Aurora^21^.

### Evidence for ultramafic-influenced venting and subseafloor serpentinization

**Fig. 5:**
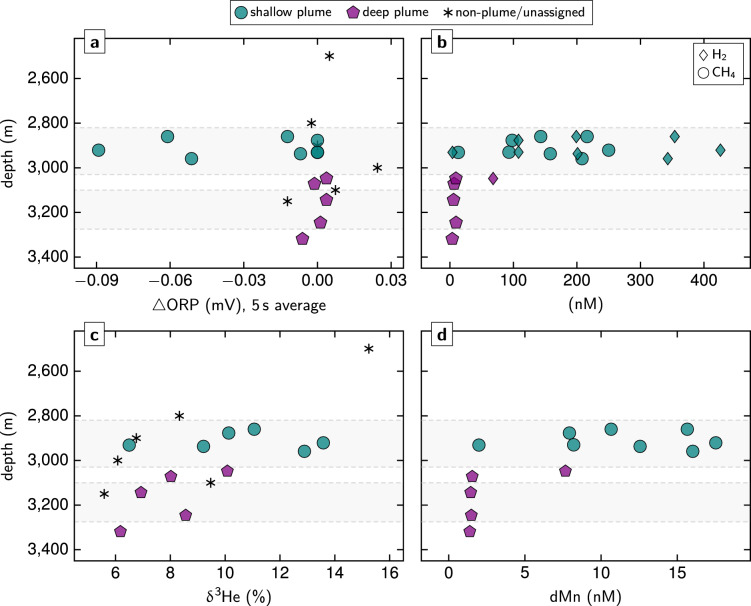
Lucky B plume sample characteristics plotted against water depth. Anomalies in ORP **(a)** correlate with high H_2_ and CH_4_ concentrations **(b)**, with δ^3^He excursions **(c)**, and with enrichments in dMn **(d)**. Gray backgrounds mark depth intervals in which our sensors detected hydrothermal anomalies in the shallow and deep plumes (Figs. 2 and 3, Supplementary Figs. S2–S4). Note considerable δ^3^He anomalies and slightly elevated CH_4_ concentrations (~ 4–10 nM) at >3,000 m depth (see also insets in Fig. 7a, b). Refer to Fig. 3 for sampling locations.

We used chemical analysis of plume water samples to constrain the nature of fluids responsible for the observed hydrothermal plumes. When plotted as a function of depth, results from our two CTD surveys reveal pronounced compositional differences between the shallow and deep plumes (Fig. 5, Supplementary Table S2; see Fig. 3 for sample locations). While both plumes carried significant above-background δ^3^He anomalies, indicative of submarine hydrothermal inputs, other chemical anomalies were considerably more enriched in the shallower plume. These samples exhibit strong enrichments in dissolved H_2_ and CH_4_, reaching concentrations of up to 426 nM and 250 nM, respectively, coinciding with the most intense ΔORP values (Fig. 5a, b). Helium isotope compositions at these depths also show pronounced excursions, with δ^3^He as high as 13.6%, and dissolved Mn (dMn) concentrations reach up to 17.5 nM—markedly less than the corresponding H_2_ and CH_4_ concentrations (Fig. 5c, d).

Although the strong chemical gradients in the shallow plume overshadow the signals from the deeper plume, both were clearly identifiable through in situ sensing (Fig. 3). Relative to background concentrations of the water column, the deep plume displays enrichments in δ^3^He of up to 9% (Fig. 5c) and enrichments in CH_4_ and dMn of up to ≤ 10 nM and ≤ 1.6 nM, respectively, even though those enrichments are not readily apparent when plotted at the scales necessary to represent the shallower plume concentrations for the same tracers (Fig. 5b, d; see also below). In contrast, dissolved H_2_ was not detectable in the deep plume.

**Fig. 6:**
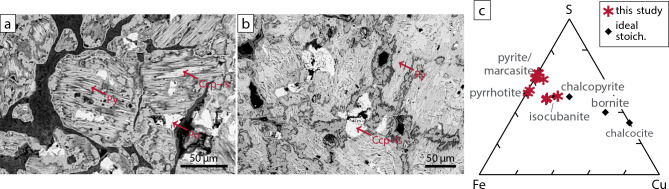
Seafloor massive sulfide mineralogy. **(a, b)** SEM images of polished thin sections. Ccp—chalcopyrite, Is—isocubanite, Po—pyrrhotite, Py—pyrite. **(c)** Sulfide compositions as molar proportions in Cu–Fe–S space as derived by energy dispersive X-ray spectroscopy. Pyrite, pyrrhotite, and phases compositionally close to isocubanite dominate the mineral assemblage. Samples recovered in dredge PS55/088^29^ (dashed line in Fig. 2a). ‘Ideal stoich.’ refers to ideal stoichiometric compositions.

As a complement to our water column analyses, we also analyzed massive sulfide chimney material recovered from the Lucky B site in 1999 (dredge PS55/088; Figs. 1c and 2a) The material is composed primarily of Fe sulfides and Cu–Fe sulfides, with chalcopyrite (CuFeS_2_), isocubanite (CuFeS_3_), pyrite (FeS_2_), and pyrrhotite (Fe_(1–*x*)_S) as the dominant phases (Fig. 6, Supplementary Table S3). The occurrence of pyrrhotite suggests precipitation under strongly reducing (low *f*O_2_) and S-depleted (low *f*S_2_) conditions—characteristics consistent with serpentinization-derived hydrothermal fluids^42,43^.

Discharge of H_2_- and CH_4_-rich fluids, as clearly indicated from the shallow plume chemical anomalies, is a well-documented feature of both high- and low-temperature ultramafic-hosted hydrothermal systems influenced by serpentinization^3,22,44,45^. Consistent with this, the observed mineral assemblage of chalcopyrite–isocubanite, pyrite, and pyrrhotite closely resembles those found in black smoker chimneys at other ultramafic-associated sites^46,47^. Although pyrrhotite formation is commonly associated with serpentinizing hydrothermal systems, its presence alone is not necessarily diagnostic of ultramafic-hosted venting. At Lucky B, however, the exceptionally high concentrations of dissolved H_2_ and CH_4_ in the overlying plume strongly support such an association.

### H_2_- and CH_4_-enriched black smoker-type venting

**Fig. 7:**
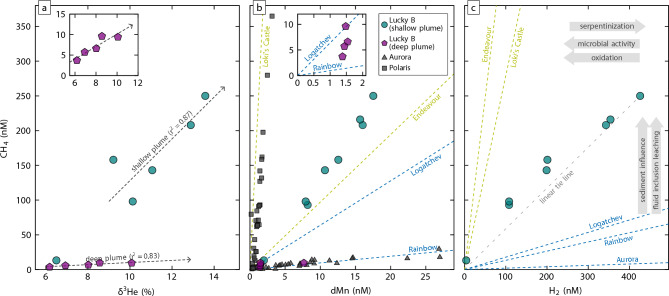
Relationships between dissolved CH_4_ and other hydrothermal species in the Lucky B plume samples. **(a)** Methane concentrations follow two distinct linear trends with δ^3^He: higher CH_4_ in the shallower plume and lower in the deep plume. Note that ranges in δ^3^He of the two plumes overlap. Inset with expanded y-axis focuses on deep plume data. Dashed lines depict linear best fits. **(b)** CH_4_/dMn values in the deep plume (inset shows samples from CTD 058) reveal low-level co-enrichments of dMn and CH_4_, similar to ratios observed at Logatchev and Rainbow. CH_4_/dMn in the shallow plume at Lucky B exceed those observed at the Logatchev and Rainbow and are also distinct from the ultramafic-influenced Arctic Aurora and Polaris sites. Instead, they resemble those from the sediment-influenced Endeavour Vent Field, suggesting a possible sediment involvement at Lucky B. For context, data from Loki’s Castle, also strongly sediment-influenced, are included as well. **(c)** In the shallow plume, CH_4_ and H_2_ concentrations correlate. The sample with the highest H_2_ concentration (426 nM) also exhibits the highest H_2_/CH_4_, indicating it is the most chemically ‘fresh’. Other samples show slightly lower H_2_/CH_4_, suggesting H_2_ loss, as indicated by a leftward shift along the dashed tie line. Compared to other high-temperature ultramafic-hosted systems like Logatchev and Rainbow, Lucky B’s shallow plume has lower H_2_/CH_4_. In systems in which sediment participates in subseafloor fluid–rock reactions, e.g., Loki’s Castle and Endeavour, CH_4_ concentrations are elevated and H_2_/CH_4_ is lower, aligning with patterns at Lucky B. Our samples from the deep plume did not contain detectable H_2_ concentrations. Sampling locations are shown in Fig. 3. Data from Logatchev, Rainbow, Loki’s Castle, and Endeavour taken from MARHYS Database v4.0^55^, with dashed lines depicting tie lines from the origin to the average values for CH_4_/dMn and H_2_/CH_4_ (samples with Mg ≤ 5 mM); Aurora data from Seewald et al.^22^ and German et al.^16^, Polaris data from Albers et al.^17^.

Relationships between CH_4_ concentrations, δ^3^He compositions, and concentrations of dMn and H_2_ provide valuable insights into subseafloor and water column processes at Lucky B (Fig. 7). Methane exhibits two distinct linear trends with δ^3^He, one corresponding to the shallow plume and the other to the deeper plume (Fig. 7a). The trend observed in the shallow plume displays elevated CH_4_ concentrations (≤ 250 nM) that align with the highest deviations in He isotope values in our dataset. This pattern likely reflects focused discharge of hot fluids from black smoker chimneys, which typically reach neutral buoyancy within an hour after discharge from the seafloor^38^. Because δ^3^He is chemically inert and unaffected by consumption^48,49^, the linear relationship demonstrates that CH_4_ also behaves conservatively in the near-field plume samples and changes in concentration are attributable to simple dilution with ambient seawater. In prior work it has been noted that over sufficiently long distances down-plume, dissolved CH_4_ can behave non-conservatively with respect to δ^3^He anomalies^50^. However, over shorter length- and timescales down-plume from a source, more akin to this study’s sample suite, it is not unusual to see dissolved CH_4_ behave conservatively with respect to δ^3^He (ref.^51^).

Concentrations of δ^3^He and CH_4_ in the deep plume are also correlated, but the slope of this correlation is considerably less steep, primarily because CH_4_ enrichments are smaller (Fig. 7a). These lower CH_4_ concentrations are unlikely to result from mixing between seawater and the same vent fluid that feeds Lucky B’s shallow plume, as the δ^3^He values in the same samples are not similarly reduced. Instead, the quasi-linear trend observed between CH_4_ and δ^3^He, together with the considerably lower CH_4_ levels, suggests dilution of fluids from a different vent source feeding the deep plume.

The presence of H_2_- and CH_4_-rich hydrothermal plumes atop a peridotite ridge is consistent with a high-temperature ultramafic-hosted vent environment, similar to systems along the Mid-Atlantic Ridge^52^. However, the shallow plume at Lucky B is unusually enriched in CH_4_ compared to those sites. Previous studies have used ratios of CH_4_ to dMn to distinguish among mafic-, ultramafic-, and sediment-influenced hydrothermal sources^14,16^. At Lucky B, CH_4_/dMn in the deep plume varies from 1.2 to 6.5, consistent with ratios observed at ultramafic-influenced high-temperature vents such as Ashadze, Rainbow, and Logatchev at which average CH_4_/dMn ranges from 0.96 to 6.4^45,53,54^ (Fig. 7b; averages determined using data from MARHYS v4.0^55^). CH_4_/dMn in the shallow plume, however, ranges from 11.3 to 14.3—well exceeding those values at Ashadze, Rainbow, and Logatchev. Higher ratios from ultramafic-influenced sites are known from Von Damm on the Mid-Cayman Rise (CH_4_/dMn = 171–331^3,56^) and the Polaris site on Gakkel Ridge (CH_4_/dMn ≤ 116^17^) but those systems vent at lower temperatures, generating metal-poor plumes that cause their CH_4_/dMn to be high.

### A sediment-influenced ultramafic-hosted system?

Among global data, the CH_4_/dMn at Lucky B plume most closely resembles that of the Endeavour Vent Field on the Juan de Fuca Ridge (Fig. 7b). At that basalt-hosted system, high concentrations of NH_3_, in addition to CH_4_, have been attributed to assimilation of sedimentary material into the underlying lithosphere, resulting in thermogenic CH_4_ production^57–59^. More recently, studies at two basalt-hosted vent sites in the Norwegian–Greenland Sea, Loki’s Castle and Jøtul, suggested the influence of similar sedimentary components there^40,60^. Both sites exhibit even higher CH_4_/dMn than Lucky B (Fig. 7b). At Loki’s Castle, these elevated ratios result from CH_4_ concentrations of 9.2 mM, unusually high for a basalt-hosted black smoker site, and low Mn concentrations of < 0.08 mM^60^. Black smoker fluids venting at Jøtul are similarly enriched in CH_4_ with up to 9.5 mM^40^. Thus, the CH_4_/dMn signature at Lucky B aligns with that of sediment-influenced systems.

A sedimentary component at Lucky B is plausible, given its proximity to the sediment-rich Greenland Shelf and Yermak Plateau (Fig. 1a). Additional support comes from PS66 dredge samples collected in 2004, which recovered chimney fragments composed of intergrown carbonate and sulfide^30^. Such carbonate formation is typically associated with the higher pH of vent fluids at sediment-influenced sites, in contrast to the low pH values, as low as 3, observed at purely ultramafic sites^45,61–63^. Though plausible, the Lena Trough—and the Lucky Ridge in particular—lacks significant volcanic eruptives, as indicated by abundant mantle rock exposure and weak magnetic anomalies^26,27^. In contrast to basalt-hosted systems, where sediments can be buried and incorporated by volcanic activity, the predominantly tectonic style of seafloor spreading at Lucky B presents a challenge for sediment incorporation into the subseafloor.

Whereas CH_4_ in vent fluids is generally believed to originate primarily from the reduction of dissolved CO_2_ in circulating fluids^64,65^, an additional CH_4_ source at Lucky B could be fluid inclusions within mafic minerals in the subseafloor. Serpentinization reactions in such inclusions can generate H_2_ which facilitates abiotic CH_4_ synthesis over geologic timescales^66^. At the mafic–ultramafic-hosted Von Damm site, Mid-Cayman Rise, CH_4_ in vent fluids is radiocarbon-dead, indicating formation within fluid inclusions long before its leaching by circulating fluids^3,56^. While C within CH_4_ from the fluid inclusions at Von Damm appears mantle-derived, seawater-derived CO_2_ may also contribute at other sites^67^. In the Lena Trough, ample tectonic activity should allow seawater penetration into the basement and formation of fluid inclusions, which may have trapped CO_2_ and generated CH_4_ that is now being leached, enhancing its concentrations in the vent fluids.

Sediment-influenced basalt-hosted systems also show distinctly low ratios of H_2_ to CH_4_ (Fig. 7c), despite high H_2_ concentrations in endmember fluids (e.g., 5.5 mM at Loki’s Castle^60^). At Lucky B, H_2_ concentrations in the shallow plume correlate well with CH_4_ (Fig. 7c), yielding H_2_/CH_4_ of 1.1–1.7. These exceed values observed in sediment-influenced sites but are considerably lower than those from the ultramafic-hosted Rainbow and Logatchev^45,54,58^. The lower H_2_/CH_4_, again, support a sediment-influenced origin for Lucky B fluids. The relatively high H_2_ levels at Lucky B are likely driven by serpentinization of the underlying ultramafic rocks^45,68^; in fact, our measured H_2_ concentrations are within range of those in non-buoyant plumes of other ultramafic-influenced vent sites^69,70^. However, H_2_ is not expected to behave conservatively during plume dispersion^50^ (see also below) so that our measured H_2_/CH_4_ of ≥ 1:1 represents conservative lower bounds. Source fluid compositions are likely richer in H_2_ relative to CH_4_, closer to the values for Aurora, Rainbow, and Logatchev (Fig. 7c). The absence of detectable H_2_ in the deep plume samples implies that these fluids were either never enriched in H_2_ or that most H_2_ had already been consumed.

In summary, endmember fluids at Lucky B likely contain elevated H_2_ and CH_4_, resulting in H_2_/CH_4_ and CH_4_/dMn intermediate between those of ultramafic and sediment-influenced sites. Lucky B may thus represent an ultramafic analogue to sediment-influenced mafic-hosted systems. Definitive confirmation of a sediment contribution will require sampling of the seafloor vent sources and analysis of NH_3_, CH_4_, CO_2_, H_2_, and stable and radiogenic C isotopes.

### Microbial carbon fixation and community composition

**Fig. 8:**
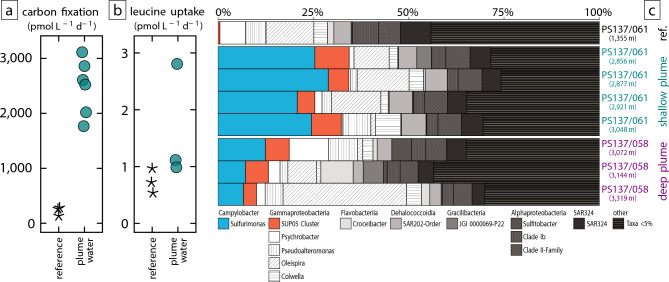
Microorganisms growing in Lucky B’s shallow plume. **(a)** Dark carbon fixation in reference background seawater and plume samples (2,876–2,921 m depth, CTD 061) measured in water incubations with ^14^C-bicarbonate tracer. **(b)** Rates of leucine incorporation into biomass in reference seawater and plume water samples (2,876 m depth, CTD 061) measured in a ^3^H-leucine assay. **(c)** Microbial diversity in reference and plume samples based on amplified 16S rRNA gene sequences. Reference samples taken at CTD station PS137/18 in 3,200 m water depth at 82°36.5796ʹN, 5°53.184ʹW.

In order to evaluate the impact of reduced compounds in the Lucky B’s shallow plume on deep-sea microbial activity, we incubated seawater samples with ^14^C-bicarbonate and ^3^H-labeled leucine (Supplementary Table S4). We assessed the assimilation by measuring the uptake of these tracers into particulate organic matter ≥0.2 µm in size. In non-plume reference samples, dark carbon fixation—measured as the uptake of dissolved inorganic carbon into microbial biomass—was < 300 pmol L^–1^ d^–1^. Contrastingly, plume samples exhibit 1.77–3.11 nmol L^–1^ d^–1^ (*n* = 2 biological replicates; Fig. 8a). This approximately tenfold increase in carbon fixation indicates that the reduced compounds in the plume water strongly stimulate chemoautotrophic microbial activity. In comparison, uptake of ^3^H-leucine, a proxy for heterotrophic activity^71^, showed a more modest increase from 0.7 pmol L^–1^ d^–1^ in non-plume background to 1–2.8 pmol L^–1^ d^–1^ in the plume (Fig. 8b). Autotrophy is hence the dominant process leading to CO_2_ fixation in Lucky B’s near-field plume^72^.

We also amplified and sequenced bacterial 16S rRNA genes from both the deep and shallow plumes. A reference sample from background seawater contained mostly heterotrophic water column bacteria, including *Flavobacteria*, *Dehalococcoidia*, *Oleispira*, and SAR324 (Fig. 8). Conversely, the plume samples showed a pronounced shift in community composition, with *Sulfurimonas* dominating the assemblage (Fig. 8c). In the deep plume, *Sulfurimonas* reached a relative abundance of 12%, whereas in the shallow H_2_-rich plume, abundances were as high as 30%. *Sulfurimonas* are likely the H_2_-oxidizing bacterium *Candidatus* Sulfurimonas pluma^73,74^. The relative abundance of SUP05 bacteria was also elevated in Lucky B’s deep and shallow plumes (3–6% and 5–9%, respectively) compared to background seawater (~ 1%). These bacteria are known as autotrophic sulfide oxidizers in oxygen minimum zones^75^ but also thrive in hydrothermal plume environments^73,74^. Prior studies suggested that *Ca.* S. pluma and SUP05 grow on H_2_ and H_2_S as electron donors, respectively^73,75^. Assuming that the microbial cell yields scale with the free energy yields from the oxidation of these electron donors, the dominance of *Ca.* S. pluma over SUP05 in Lucky B’s plumes suggests that the concentration of bioavailable H_2_S is lower than that of H_2_. This interpretation is supported by the metal enrichments in the plumes (Fig. 5d), which likely cause the precipitation and removal of H_2_S from solution. In the deep plume, high abundances of *Ca.* S. pluma may have effectively consumed H_2_, reducing concentrations below our detection limits—consistent with laboratory experiments and in situ measurements^74^. On the other hand, the δ^3^He anomalies and the elevated CH_4_ concentrations persist (Fig. 7a), indicating that these tracers exhibit greater conservativeness in the plumes. Methanotrophs are consistently rare across all samples, with no detectable increase in either plumes. This suggests that CH_4_ is not a major near-field energy source at Lucky B, consistent with its approximately conservative behavior in our samples (Fig. 7).

Hydrothermal venting at Lucky B hence facilitates chemoautotrophic carbon fixation in the Arctic Ocean. Notably, very similar microbial community compositions have been observed in the plumes of the only two other well-studied vent sites in the high Arctic, Aurora and Polaris, which are also dominated by *Ca.* S. pluma and SUP05^73,74^. Both Aurora and Polaris emit fluids that are also enriched in H_2_, and putatively H_2_S, but both are located on basaltic seafloor and H_2_ production is inferred to result from serpentinization deeper in the subseafloor^16,17,22^. Thus, *Ca.* S. pluma and SUP05—microbes that utilize H_2_ and H_2_S—appear to be a consistent feature in hydrothermal plumes of serpentinization-influenced hydrothermal plumes along slow-spreading ridges^73,74^, and their relative abundances offer insights into the plume’s chemical compositions.

## Conclusions and implications

Hydrothermal circulation at Lucky B mobilizes carbon within the subseafloor, facilitating the formation of CH_4_ and its release into the overlying water column. At the seafloor, the discharge of highly reducing hydrothermal fluids promotes the precipitation of carbonate minerals and supports abundant hydrothermalism-associated fauna, with both processes contributing to carbon fixation at the seafloor. Additionally, active microbial chemosynthesis within Lucky B’s hydrothermal plume captures further carbon in the water column. The interaction of an ultramafic seafloor—providing substantial reducing power through serpentinization—with a carbon source, whether sedimentary or magmatic in origin, drives complex and multifaceted influences on the oceanic carbon cycle at Lucky B.

Each of the three vent systems studied in detail along the high Arctic spreading centers has yielded unexpected findings. Aurora and Polaris on Gakkel Ridge were initially considered mafic-influenced black smoker systems until Aurora was shown to be strongly influenced by serpentinization^16,21^ and Polaris was revealed to be an intermediate-temperature, hybrid mafic–ultramafic system^17^. With the discovery of Lucky B, we identified yet another distinct type of venting in the Arctic Ocean. Although situated on ultramafic seafloor, Lucky B exhibits exceptionally high CH_4_/dMn rather characteristic of sedimentary-influenced systems than typical ultramafic-hosted black smoker systems. This highlights the remarkable geological diversity of H_2_-producing hydrothermal systems along the ultraslow-spreading high Arctic ridges, where at least seven additional hydrothermal vent sites remain unexplored^18^. Similar diversity may exist on the comparably ultraslow-spreading Southwest Indian Ridge, where hydrothermal plume signals were first detected even before those on Gakkel Ridge^76^. Yet, nearly three decades later, only two high-temperature vent fields there have been definitively located^77,78^. Given the logistical challenges of assessing the ice-covered Gakkel Ridge and the remoteness of the Southwest Indian Ridge from major oceanographic research bases, the serpentinization-influenced Lucky B vent field offers a rare, more accessible natural laboratory for future interdisciplinary research.

## Methods

### Water column surveys

#### CTD rosette

The dispersion of the hydrothermal plume was tracked using a Sea-Bird Scientific SBE 911plus CTD (conductivity–temperature–density) unit with additional particle and redox sensors^33^. All sensors were monitored in real-time and recorded time intervals were postprocessed to 1 s. Due to technical problems with the turbidity sensor, beam transmission detected by the transmissiometer was used as a proxy for turbidity. The CTD position relative to the seafloor was monitored using an ultrashort baseline navigation system. The operational strategy was similar to that described in German et al.^16^ and Albers et al.^17^: the ship was allowed to drift with sea ice and deployments of the CTD, OFOBS, and NUI (see below) were planned to pass as close as possible to the inferred vent sites by using a sea ice drift model^33^. After each cast, anomalies in oxidation–reduction potential (ΔORP) were calculated from redox sensor data as the deviation of two consecutive measurements. To account for outliers and for visualization, data were smoothed with moving 5- or 30-point averages. Potential densities were computed using the TEOS-10 (Thermodynamic Equation of Seawater – 2010) standard, implemented using the Gibbs Seawater (GSW) Oceanographic Toolbox^79^ in Python.

MAPRs (Miniature Autonomous Plume Recorder) from the National Oceanographic and Atmospheric Association/Pacific Marine Environmental Laboratories^80^ were attached directly to the NUI vehicle and OFOBS device (see below) during seafloor imaging station (see ‘Evidence for ultramafic-influenced venting and subseafloor serpentinization’) to record hydrothermal signals during seafloor imaging; they were set to record data in 5 s intervals. Anomalies in ΔORP from were derived as described above. Potential temperature was calculated from the MAPR recordings following Millero and Poisson^81^; anomalies in potential temperature (Δθ) relative to ambient seawater were calculated by subtracting the recorded temperatures from 2nd or 3rd order polynomial functions that fit the seawater above and below the hydrothermal anomalies.

#### Nereid Under Ice plume sensing

A key component of our search strategy at Lucky B was the deployment of the lightly tethered hybrid autonomous underwater vehicle (AUV)/remotely operated vehicle (ROV) Nereid Under Ice (NUI). NUI uses a combination of battery power and a single unarmored fiber optic cable to be able to move laterally through an ice-covered ocean, from ice–ocean interface down to the seafloor, while maintaining real time high bandwidth communications to operators aboard ship. An advantage of the vehicle is that it can operate in (micro-tethered) AUV mode while surveying the ocean water column and seafloor and then convert to an ROV, by opening its forward-bay doors, to conduct more detailed seafloor imaging and sampling operations^82^. During its water column survey, the primary sensors utilized aboard NUI were the same package routinely used for AUV-based plume surveys (CTD, optical backscatter, and ORP sensors; German et al.^83^) augmented with a Franatech in situ METS Methane Sensor. The METS sensor was not calibrated, hence providing relative enrichments rather than absolute concentrations of dissolved CH_4_ in the water column. Internal precision for the positioning of NUI during each of the three stages of its survey work portrayed was achieved using a downward looking Doppler Velocity Logger that sustained bottom-lock during the plume-height, mapping, and geological transect operations. Accuracy in the positioning of the vehicle was obtained by attaching an ultrashort baseline beacon to the vehicle that allowed us to determine NUI’s position at depth via the R/V Polarstern’s Posidonia navigation system.

The water column ‘sensing’ survey was carried out at water depths between ~ 3,200–3,300 m on the western slope of Lucky Ridge, following the information from previous expeditions^29,30^. NUI executed an approximately lateral zig-zag track line from ~ 81°22.3’N to ~ 81°21.6’N, repeatedly approaching and retreating from the Lucky Ridge.

Following completion of the ‘sensing’ survey, NUI was directed to undertake a higher resolution ‘mapping’ survey at ~ 50 m altitude over features of interest picked from submarine morphology within the region of highest CH_4_ anomalies. A series of five track lines spaced at 75 m and roughly orthogonal to the strike of the Lucky Ridge were followed to multibeam coverage of the seafloor, using the vehicle’s 500 kHz Norbit WBMS multibeam system, and yielding a grid at 1 m resolution.

### Plume chemistry

The CTD was integrated with a SBE 32 Carousel Water Sampler with 22 to 24 Niskin bottles, half of which had metal springs and the other half had silicone internal fittings, that allowed for real-time water sampling of the hydrothermal plume and background seawater. Upon recovery of the CTD–water sampler unit on deck, seawater and plume water samples were drawn for shipboard and shore-based analyses. Pairs of Niskin bottles containing water samples from the same water depths were sampled for He isotope analyses and H_2_ and CH_4_ concentrations before being sampled for dissolved metals. All samples taken for post-cruise investigations were stored at 4 °C until further analysis.

Samples for He isotopic compositions were collected in gas tight copper tubes without exposure to atmospheric air. Post-cruise analyses took place in the Noble Gas Laboratory at the Institute for Environmental Physics, University of Bremen. Samples were pre-processed with an ultra-high vacuum gas extraction system and subsequent analysis of the noble gas isotopes was conducted with a fully automated ultra-high vacuum mass spectrometric system equipped with a two-stage cryogenic system and a quadrupole and a sector-field mass spectrometer, after transfer into a glass ampoule kept at liquid nitrogen temperature via water vapor. The precision for ^3^He/^4^He was ± 0.4% or better. For details, see Sültenfuß et al.^84^.

To determine H_2_ concentrations, 60 mL syringes were filled with 40 mL water from the CTD rosette. A 10 mL (1 atm) headspace of H_2_-depleted synthetic air (N_2_:CO_2_ of 80:20) was added to the syringes and samples were warmed up for 1 h to reach room temperature. The syringes were shaken on a vortexer for 1 min to transfer gas into the headspace. The H_2_ concentration in the gas phase was measured with a Peak Performer gas chromatograph equipped with a reducing compound photometer (Peak Laboratories) and a 200 µL sample loop; the carrier gas was synthetic air (Alphagaz 1 Luft, Air Liquide). Dissolved CH_4_ concentrations were determined by gas chromatography using a Hewlett Packard 5890 II gas chromatograph equipped with 6-foot 5 Å molecular sieve column and a flame ionization detector following headspace extraction of 20 mL samples drawn into 60 mL plastic syringes. Dissolved H_2_ and CH_4_ concentrations were calculated by assuming quantitative transfer of dissolved gas into the headspace. The precision of technical replicates for both analyses was 5%.

Water samples for dissolved metal analyses were filtered (0.45 µm), acidified with ultrapure HNO_3_ to a pH of ~ 1.7, and stored in acid-cleaned Sarstedt™ tubes. Trace metal concentrations were determined by high-resolution inductively-coupled plasma mass spectrometry at Texas A&M University. Samples were preconcentrated (× 15) via an offline seaFAST pico system^85,86^, and Mn was quantified using a matrix-matched standard curve. Briefly, a 3 mL aliquot of sample was injected into a seaFAST pico extraction system, buffered inline to pH ~ 6.3, and loaded onto a Nobias-chelate PA1 resin column. Ultrahigh purity water (Milli-Q) was passed over the column to remove sea salts before being back-eluted with 200 µL 10% (v/v) HNO_3_ (Optima). Samples were then analyzed on a Thermo Element XR in the R. Ken Williams Radiogenic Isotope Geoscience Laboratory. External reference materials GD and GSC were analyzed concurrently to confirm methods produced dissolved metal recoveries within consensus values.

### Seafloor observations

Upon completion of the mapping component of the NUI dive, the vehicle was driven to the seafloor under real-time ROV pilot control to conduct a geological transect upslope across the terrain that had just been mapped in detail. For this final component of the NUI dive, imaging was conducted using a combination of a fixed mount Rayfin HDE-GigE-6000-DBH13-LO (1920 × 1080 pixels) camera^82^. The same sensors used for plume detection during the water column component of our NUI Dive at Lucky B continued to be recorded throughout the mapping and geological transect components of the same multi-modal dive.

Seafloor observations at Lucky B were also obtained, after NUI operations were complete, using the towed underwater camera system Ocean Floor Observation and Bathymetry System (OFOBS) of the Alfred Wegener Institute, Helmholtz Centre for Polar and Marine Research^87^. OFOBS was equipped with a HDTV camera, a high-resolution photo camera (iSiTEC, CANON EOS 5D Mark III), two strobe lights (iSiTEC UW-Blitz 250), three laser pointers at a distance of 50 cm from each other to estimate the size of seafloor structures, and a beacon communicating with an ultrashort baseline navigation system to track its position during deployments. The positioning system did, however, not function reliably throughout the deployment; missing segments were reconstructed by linearly interpolating the first and last recorded positions, using the ship’s position as a reference. The depth recordings remained unaffected by the malfunctioning and were used to validate the interpolated OFOBS positions. The feed from the HDTV camera was transmitted to the ship in real-time; photos were taken in regular intervals and on demand. OFOBS also gathered sidescan data over ~ 100 m swath width, using a EdgeTech 2205 AUV/ROV multiphase echosounder.

### Analysis of hydrothermal precipitates

Analysis of massive sulfides samples recovered in 1999 (dredge PS55/088, samples 3 and 5, of cruise PS55^29^) was conducted at University of Münster. Samples were cut and polished for optical and scanning electron microscope (SEM) investigations and analyzed using a JEOL 6610 SEM. Individual mineral phases across the samples were measured using multiple spot analyses (3–5 per phase) with the built-in energy dispersive X-ray (EDX) analyzer to obtain representative mineral compositions.

### Measurements of microbial activity

The assessment of microbial autotrophic and heterotrophic activities in seawater samples was conducted through the application of bicarbonate and leucine incorporation-based methodologies, respectively^88^. Rates of dark CO_2_ fixation (DCF) were assessed using the methodology described by Wegener et al.^74^, with the following modifications: water samples were collected with sterilized 50 mL DGA glass syringes (Sigma-Aldrich, Merck), and plume seawater were incubated for 6 h after the injection of sodium bicarbonate [14C] (55 mCi/mM of specific activity; Hartmann Analytic). The measurement of microbial heterotrophic activity was conducted, as described in previous deep-sea studies^89^, by addition of L-[4,5-3H]-Leucine (3H-Leu, 100 Ci mmol-1 of specific activity; Hartmann Analytic) at a final concentration of 10 nM, followed by a 12 h incubation period.

### Extraction of genomic DNA and amplification and sequencing of 16S rRNA genes

For the 16S rRNA gene analysis, 10 L seawater from the Niskin bottle were filtered with a peristaltic pump at 2 °C on 0.22 µm pore size Sterivex filters. The filters were stored at –20 °C until extraction. In the home laboratory the Sterivex filters were cut into small pieces and transferred into bead-beating tubes (Qiagen). Microbial DNA was extracted with a DNeasy PowerWater Kit (Qiagen, Venlo, Netherlands) following manufacturer’s recommendations. For gene sequencing, the variable V3-V4 region of the 16S rRNA gene was amplified using the primer pair 341 F (5’-CCTACGGGNGGCWGCAG-3’) and 785R (5’-GACTACHVGGGTATCTAATCC-3’). Sequencing was performed at Max Planck-Genome-centre Cologne for sequencing using a NextSeq 2000 platform (Illumina, San Diego, CA, United States). Per sample, 2 × 300 pb paired end reads were produced. The raw data (fastq files) was processed following the Dada2 pipeline in R^90^. Bacterial primers using the cutadapt tool v1.9^91^. For quality control, the forward and reverse read profiles were plotted, and filter, trim, and trunc values were obtained. This analysis resulted in a 62% read retention. Error rates were plotted and the sample inference algorithm applied to the filtered and trimmed sequence data. From the denoised sequences forward and reverse reads were merged resulting in the amplicon sequence variant (ASV) table. The chimera were removed using dada2^90^. A track reads table was constructed, in which input and output of reads after every step in the pipeline were checked. The taxonomic assignment of ASVs was done with the SILVA database, v138 was used. Data was visualized with the phyloseq package in R. Chloroplasts, Mitochondria, Eukaryota, and Archaea were excluded and bar plots were produced in R with the fantaxtic (Teunisse, 2022, https://github.com/gmteunisse/fantaxtic) and the ggplot2 (Wickham, 2014, https://github.com/tidyverse/ggplot2) packages.

## Supplementary Information

Supplementary Information 1.

Supplementary Information 2.

Supplementary Information 3.

Supplementary Information 4.

Supplementary Information 5.

## Data Availability

The data presented in this study are included in this article’s Supplementary Information files and/or have been deposited at the PANGAEA database, via datalinks associated with the cruise report at https://www.pangaea.de/?q=PS137. Bacterial 16S rRNA genes have been uploaded to the National Center for Biotechnology Information (NCBI, https://www.ncbi.nlm.nih.gov/) database and is accessible via the BioProject ID PRJNA1276466. Additional data collected during PS137 aboard R/V Polarstern in 2023^33^ are also available at PANGAEA.
