# Supplementary material for: Enigmatic H2- and CH4-rich hydrothermal plumes at the ultramafic-hosted Lucky B site, 81°N on Lena Trough, Arctic Ocean
Source: Sci Rep. 2025 Oct 14;15:35912. doi: 10.1038/s41598-025-19746-5 (PMC12521595; doi:10.1038/s41598-025-19746-5)
Supplement: Supplementary file 1 — Supplementary Information 1. [file 41598_2025_19746_MOESM1_ESM.pdf]

Supplementary Information to

Enigmatic H_2- and CH_4-rich hydrothermal plumes at the ultramafic-hosted Lucky B site, 81°N on Lena Trough, Arctic Ocean

Elmar Albers[1,†,*], Felix Genske[2], Jeffrey S. Seewald[3], Maren Walter[4,5], Jonathan Mette[4], Gunter Wegener[5,6], Massimiliano Molari[6,‡], Christopher Klaembt[6,7], Luigi Gallucci[6], Tea Isler[8], Lilian Böhringer[9], Jessica N. Fitzsimmons[10], Shelby Gunnells[10], Vera Schlindwein[7,8], Christopher R. German[1]

[1]Department of Geology & Geophysics, Woods Hole Oceanographic Institution, USA
[2]Institute for Mineralogy, University of Münster, Germany
[3]Department of Marine Chemistry & Geochemistry, Woods Hole Oceanographic Institution, USA
[4]Institute of Environmental Physics, University of Bremen, Germany
[5]MARUM – Center for Marine Environmental Sciences, University of Bremen, Germany
[6]Max Planck Institute for Marine Microbiology, Germany
[7]Faculty of Geosciences, University of Bremen, Germany
[8]Section of Geophysics, Alfred Wegener Institute, Helmholtz Centre for Polar and Marine Research, Germany
[9]Deep-Sea Ecology and Technology, Alfred Wegener Institute, Helmholtz Centre for Polar and Marine Research, Germany
[10]Department of Oceanography, Texas A&M University, USA

*Correspondence: elmar.albers@awi.de
[†]now at: Section of Geophysics, Alfred Wegener Institute, Helmholtz Centre for Polar and Marine Research, Germany
[‡]now at: IMC International Marine Centre, Oristano, Italy

This document contains:

- Supplementary text
- Supplementary Figures S1 through S6 and their captions
- Captions to Supplementary Tables S1 through S4
- References

Supplementary text

Operational strategy: Plume detection and seafloor observations in an ice-covered ocean

This section outlines the sequence of operations during expedition PS137 that led to the discovery of ongoing hydrothermal activity at Lucky B.

We arrived on site just a few days before the end of expedition PS137, under logistical constraints that required all under-ice deep submergence operations with NUI to be completed at least 72 h prior to final departure. Consequently, NUI operations were limited to a single, multi-modal dive. This dive was designed to progress from water column surveys to seafloor mapping to diving to the seafloor to conduct an ROV-based geological transect—initiated immediately upon arrival on station.

This compressed schedule proved suboptimal, as it required us to dive *before* identifying consistent non-buoyant plume depths or establishing a point of closest approach to the source, which would normally be informed by CTD tow-yo surveys. Instead, our initial AUV-mode water column surveys targeted the previously reported plume depth of ~3,250 m[1]. This depth was later confirmed to coincide with the deeper of the two non-buoyant plumes detected in subsequent CTD tow-yo operations.

NUI began its mission in 'sensing' mode at depths between ~3,200–3,300 m, executing a lateral zig-zag survey from ~81°22.3′N to ~81°21.6′N, repeatedly approaching and retreating from the western slope of Lucky Ridge and passing over the locales where massive sulfides had been dredged during earlier expedition[1,2] (Fig. 2a). Hydrothermal anomalies were detected via NUI *in situ* sensors, with the strongest signals detected as the vehicle approached the seafloor at ~3,200 m near 81°22.3′N and, again, near 81°21.9′N (Fig. 2a; Supplementary Fig. S1). Switching to 'mapping' mode, NUI descending to a constant altitude of ~50 m above the seafloor (Fig. 2a). Sensor data again indicated hydrothermal anomalies concentrated in the eastern portion of the survey area. In ROV mode, NUI conducted a west–east geological transect, ascending upslope from ~3,380 m to intersect the locations of most pronounced water column anomalies (Fig. 2a).

Informed by these data, we hypothesized that any hydrothermal plume situated below but adjacent to the Lucky Ridge summit would likely be influenced by topographic steering[3]. Accordingly, we conducted two CTD tow-yos designed to follow along the ~3,250±50 m contour, where NUI had observed the strongest anomalies (Fig. 2a). CTD 058 started directly north of the northernmost anomalies, near 81°22.3′N, and immediately intercepted two distinct non-buoyant plumes: (i) at 2,850–3,000 m depth, i.e., at a shallower depth than the NUI survey had been conducted, and (ii) a deeper one at ~3,100–3,200 m (Fig. 3, Supplementary Fig. S2). The shallower plume was only observed during the initial downcast, while we migrated out of the deeper plume south of ~81°22.2′N as ice drift diverted the ship—and, hence, the CTD rosette—away from the target trackline (Fig. 2a). As soon as we could maneuver the CTD back close to the 3,250 m contour, near 81°21.9′N, the deep plume signal was reacquired and persisted to the end of the cast near 81°21.7′N (Fig. 3).

To further investigate the shallower plume intercepted during the initial downcast of CTD 058, R/V Polarstern was repositioned northward, deploying CTD 061 to follow the same ~3,250 m depth contour, heading south toward the CTD 058 starting point (Fig. 2a). Ice drift again deflected the ship and CTD slightly upslope, but hydrothermal signals appeared near 81°22.8′N, where a distinct plume was detected at 2,800–3,000 m (Fig. 3). This plume persisted throughout the remainder of the cast (Fig. 3, Supplementary Fig. S3).

Building on the insights from the NUI and CTD surveys, our final operation employed the OFOBS deep-tow system to follow the ~3,250±50 m depth contour. The survey approached the seafloor even further north of the northernmost shallow plume detection locale (Fig. 2a). However, continued ice drift again pushed both the ship and OFOBS system eastward, placing much of the survey along the shallower 3,100 m contour.

Further information is available in the R/V Polarstern expedition PS137 cruise report[4].

Supplementary figures

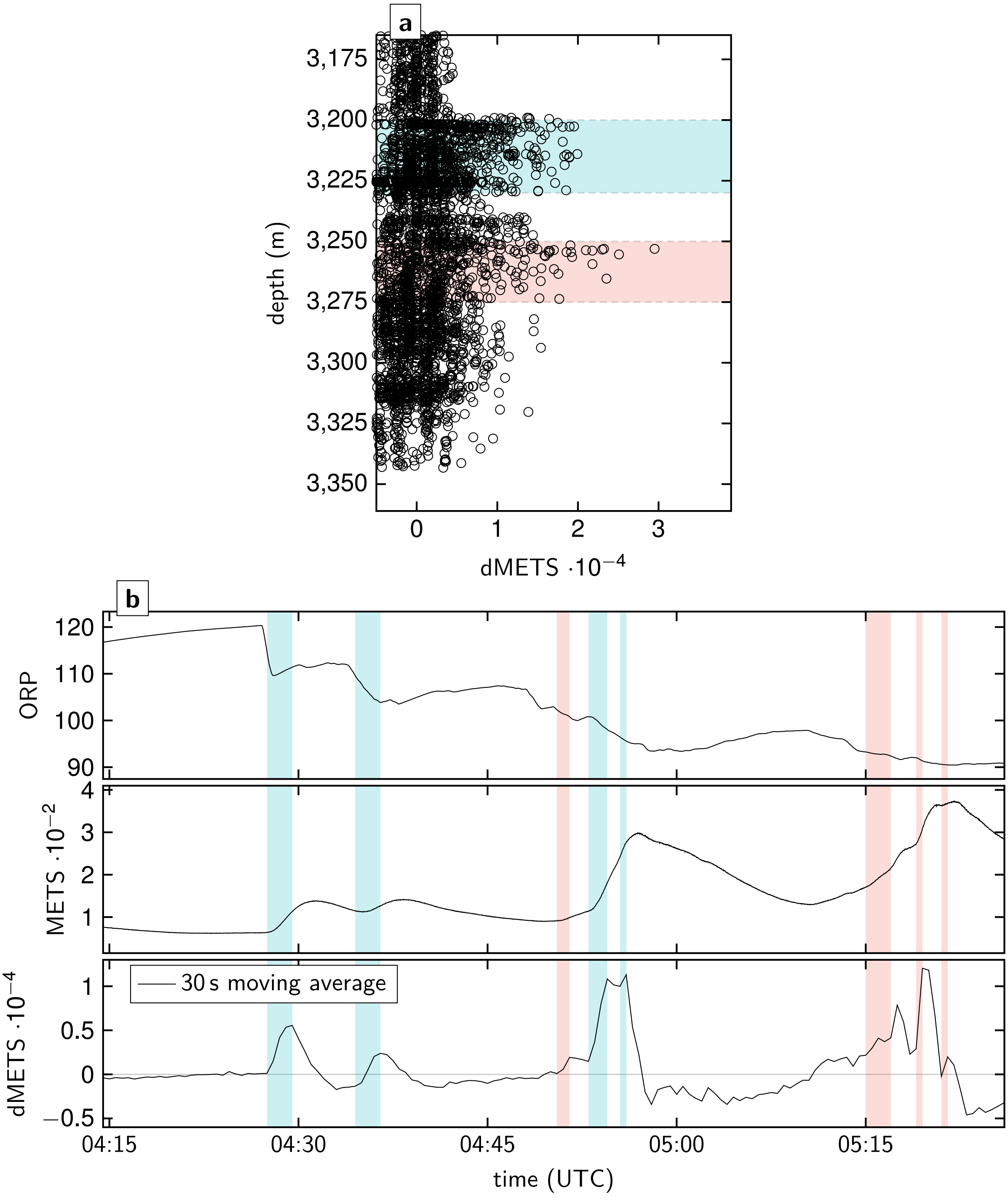

Supplementary Figure S1: Sensor readings from NUI's mapping survey (station PS137/047; cf. Figure 2a). **(a)** Vertical distribution of dMETS in the water column. When projected against depth, two clusters of CH_4 enrichment above background are apparent, at 3,200–3,225 m and 3,250–3,275 m. dMETS was calculated from METS values as the deviation of two consecutive measurements, here shown as 30 s moving averages; increases in dMETS represent CH_4 enrichments. **(b)** Time-series data of ORP, METS, and dMETS. ORP data recorded by a NUI-attached MAPR. Colored shading marks interceptions with CH_4 enrichments, as shown in (a).

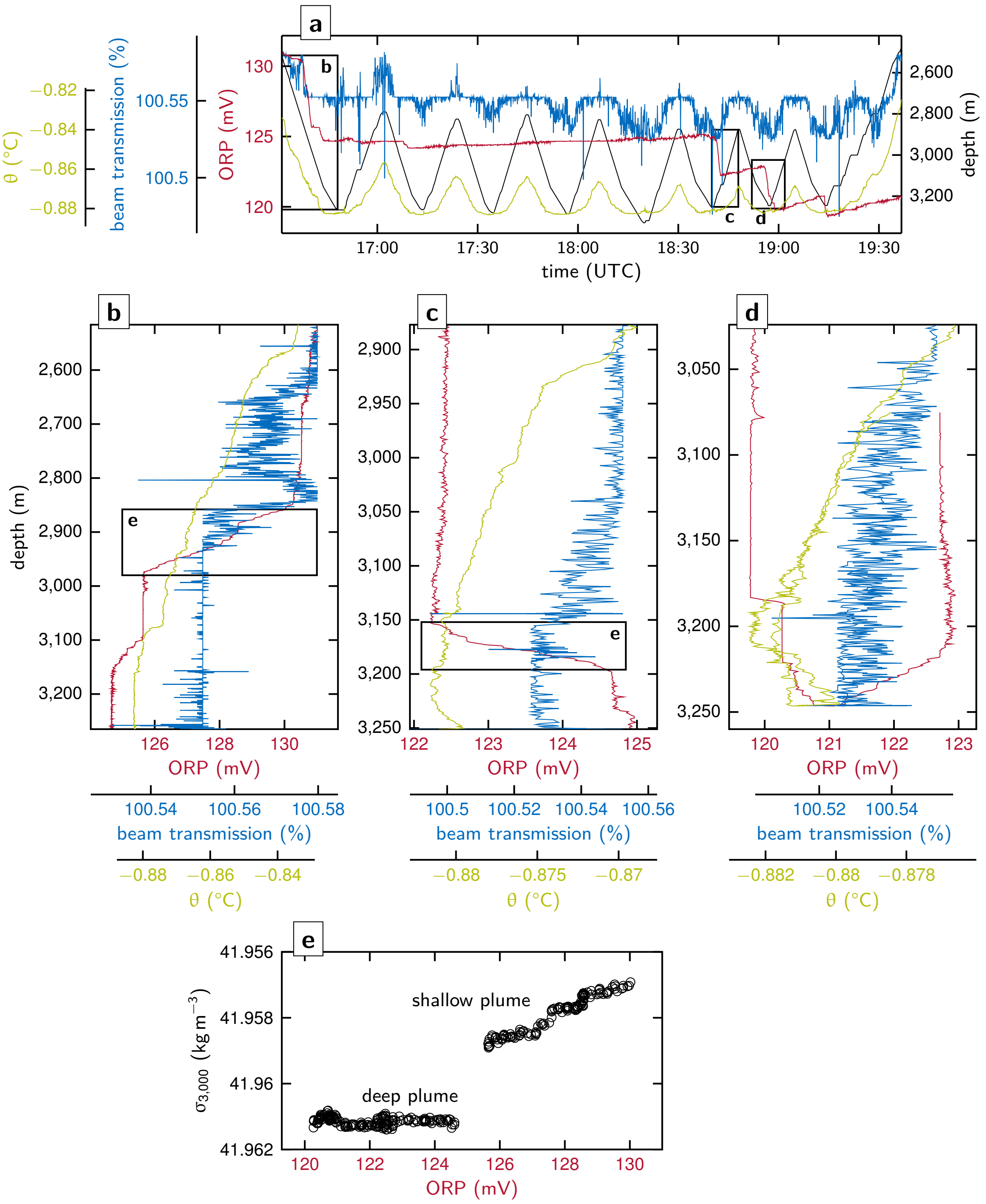

Supplementary Figure S2: Sensor readings at CTD station PS137/058 (cf. Figure 2a). **(a)** Time-series data for depth, ORP, beam transmission, and potential temperature, θ, for the entire cast, from the initial descent below ~2,500 m to recovery. **(b–d)** Close-ups of locations marked as black rectangles in (a), reprojected as vertical profiles of ORP, beam transmission, and θ. Sensor signals in (b) were recorded in Lucky B's shallow plume and those in (c) and (d) in the deep plume. Note the different ranges of the y-axes. **(e)** Individual ORP readings from single down- and upcasts shown in (b) and (c) plotted against potential density anomaly, $\sigma_{3,000}$, relative to a reference pressure of 3,000 dbar. Mean *in situ* densities,

ρ, in the shallow and deep plumes were 1041.84 kg m^{-3} (±0.16 SD; n = 141) and 1043.02 kg m^{-3} (±0.06 SD; n = 202), respectively.

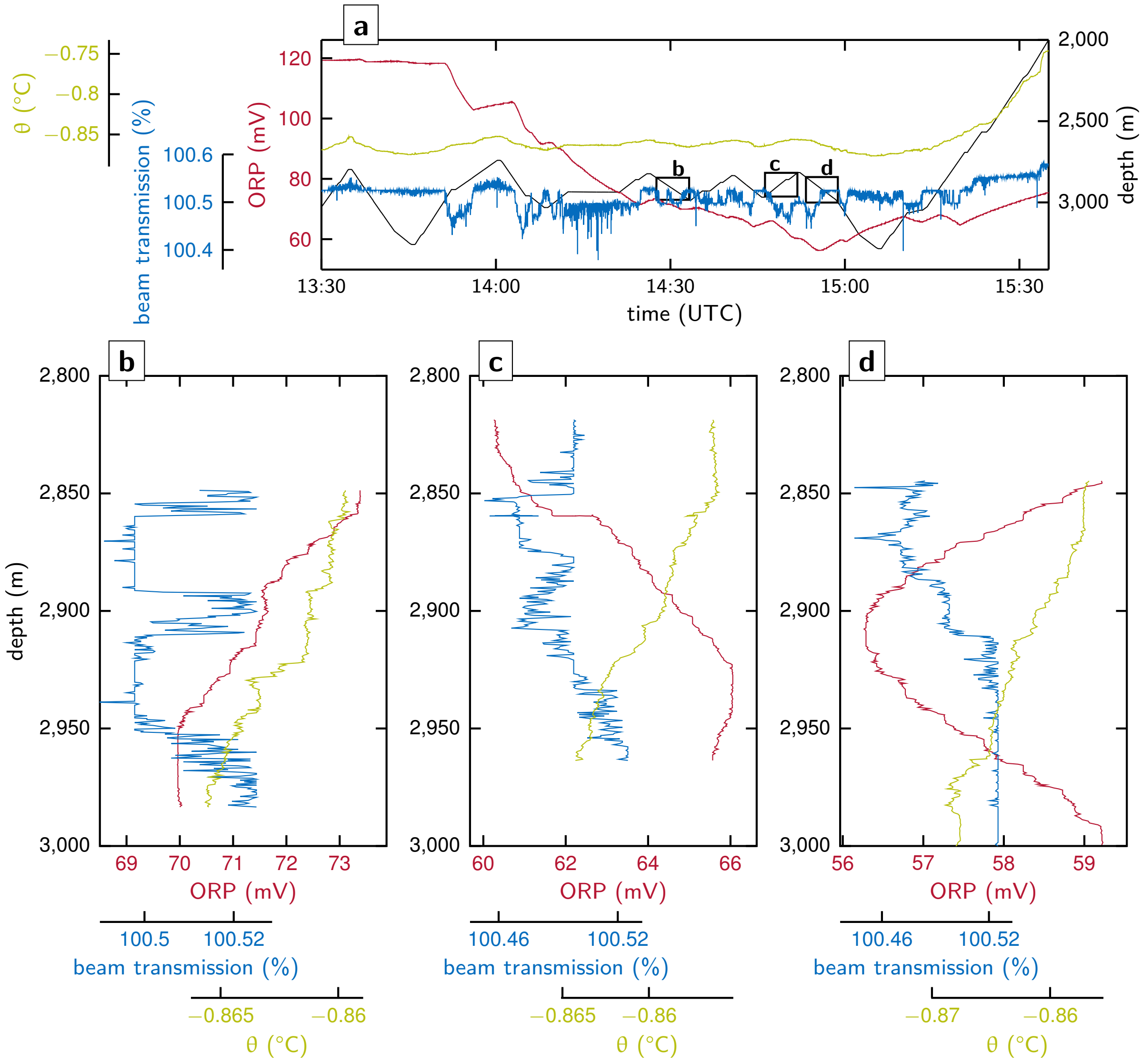

Supplementary Figure S3: Sensor readings at CTD station PS137/061 (cf. Figure 2a). **(a)** Time-series data for depth, ORP, beam transmission, and potential temperature, θ, for the entire cast, from the initial descent below ~2,000 m to recovery. **(b–d)** Close-ups of locations marked as black rectangles in (a), reprojected as vertical profiles of ORP, beam transmission, and θ, showing sensor signals from Lucky B's shallow plume.

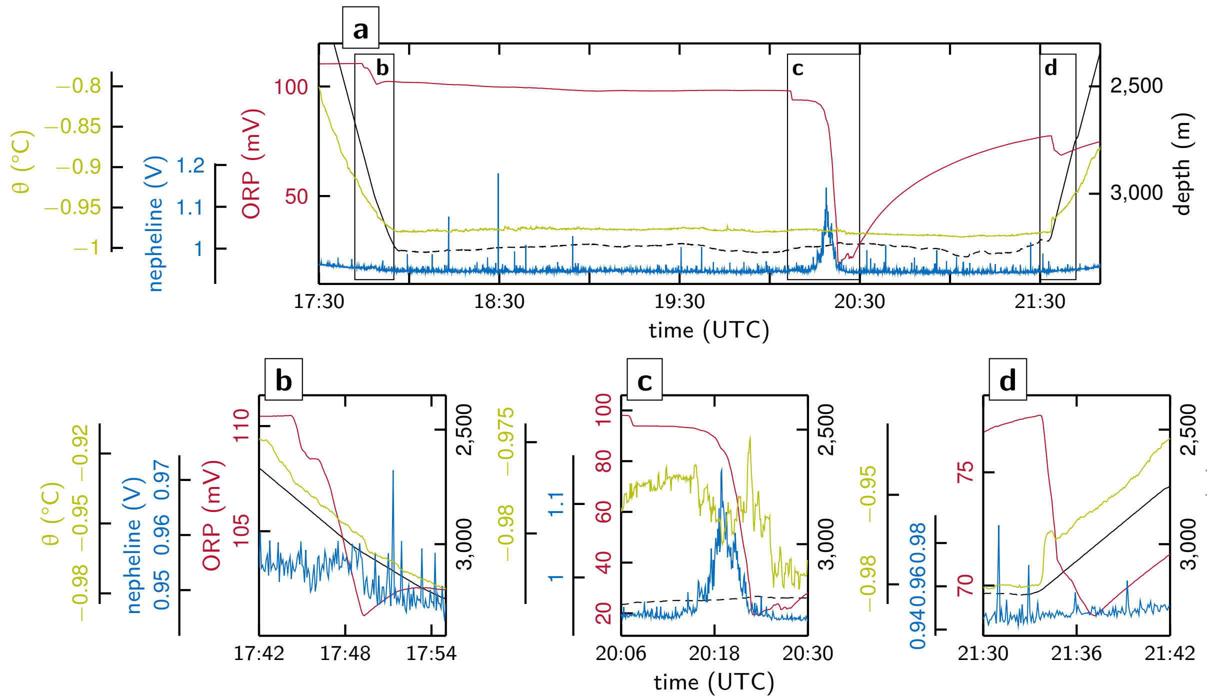

Supplementary Figure S4: Sensor readings during OFOBS station PS137/062 (cf. Figure 2a). **(a)** Time-series data for depth, ORP, nepheline, and potential temperature, θ, recorded by an OFOBS-attached MAPR. **(b–d)** Close-ups of locations marked in (a) showing sensor signals from Lucky B's shallow plume (b) and those in (c) and (d) in the deep plume. Solid black track lines mark lowering of OFOBS to and heaving the device from the seafloor, dashed lines indicate its position directly above the seafloor.

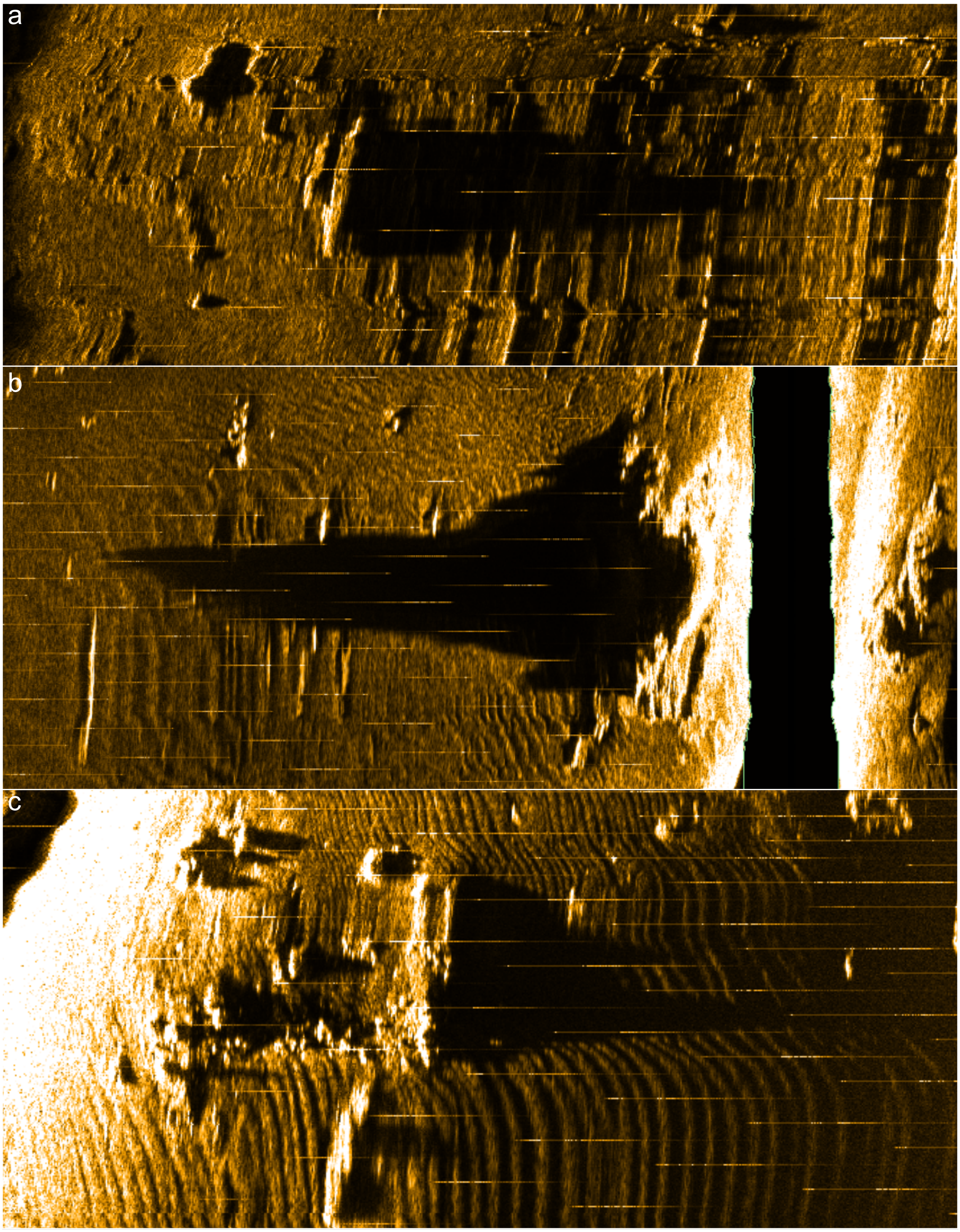

Supplementary Figure S5: Chimney structures imaged by sidescan sonar during OFOBS station PS137/062 (cf. Figure 2a). See Supplementary Table S1 for locations and calculated chimney heights; panels (a) through (c) correspond to indices 1 through 3 in Table S1.

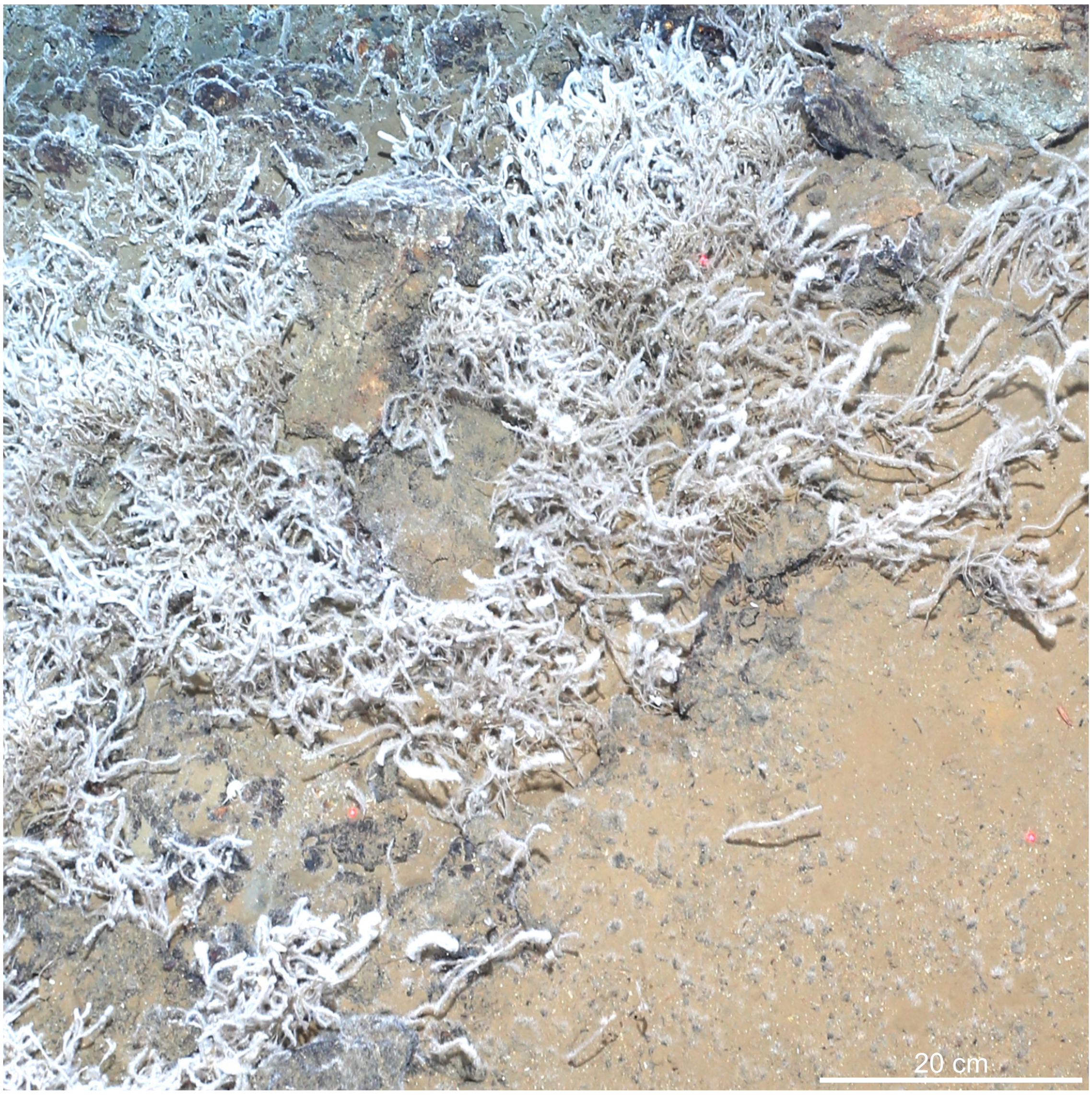

Supplementary Figure S6: Close-up of the vent fauna shown in Figure 4f. The white fibrous organisms are likely tubeworms covered in chemosynthetic bacteria, resembling siboglinid tube worms and microbial filaments found at the nearby Jøtul and Loki's Castle sites[5,6]. No physical specimens could, however, be collected during expedition PS137 to validate the seafloor images.

Supplementary tables

Supplementary Table S1: Locations and heights of hydrothermal chimney structures detected during OFOBS station PS137/062. Corresponding sidescan sonar images are shown in Figure 2 and Supplementary Figure S5.

Supplementary Table S2: Plume water chemistry of samples collected during CTD stations PS137/058 and PS137/061.

Supplementary Table S3: EDX data of massive sulfides from dredge PS55/088 (R/V Polarstern expedition PS55[2]), given in mol%.

Supplementary Table S4: Incubation results on carbon uptake rates in Lucky B's shallow plume (station PS137/061) and reference Arctic seawater (station PS137/018).

References

1 Budéus, G. & Lemke, P. The Expeditions ARKTIS-XX/1 and ARKTIS-XX/2 of the Research Vessel "Polarstern" in 2004 in *Berichte zur Polar- und Meeresforschung (Rep. Polar Marine Res.)* **544**, 1-242 (Alfred Wegener Institute for Polar and Marine Research, 2007). https://doi.org/10.2312/bzpm_0544_2007

2 Jokat, W. The Expedition ARKTIS-XV/2 of "Polarstern" in 1999 in *Berichte zur Polarforschung (Rep. Polar Res.)* **368**, 1-128 (Alfred Wegener Institute for Polar and Marine Research, 2000).

3 German, C. R. et al. Topographic control of a dispersing hydrothermal plume. *Earth Planet. Sci. Lett.* **156**, 267-273 (1998). https://doi.org/10.1016/s0012-821x(98)00020-x

4 Schlindwein, V. The Expedition PS137 of the Research Vessel POLARSTERN to the Arctic Ocean in 2023 in *Berichte zur Polar- und Meeresforschung = Rep. Polar Marine Res.* **781** (eds. Bornemann, H. & Amir Sawadkuhi, S.) 1-127 (Alfred-Wegener-Institut Helmholtz-Zentrum für Polar- und Meeresforschung, 2023). https://doi.org/10.57738/bzpm_0781_2023

5 Bohrmann, G. et al. Discovery of the first hydrothermal field along the 500-km-long Knipovich Ridge offshore Svalbard (the Jøtul field). *Sci. Rep.* **14**, 10168 (2024). https://doi.org/10.1038/s41598-024-60802-3

6 Pedersen, R. B. et al. Discovery of a black smoker vent field and vent fauna at the Arctic Mid-Ocean Ridge. *Nat. Commun.* **1**, 126 (2010). https://doi.org/10.1038/ncomms1124
